# A metabolic scenario for the evolutionary origin of peroxisomes from the endomembranous system

**DOI:** 10.1007/s00018-013-1424-z

**Published:** 2013-07-25

**Authors:** Toni Gabaldón

**Affiliations:** 1 Bioinformatics and Genomics Programme, Centre for Genomic Regulation (CRG), Dr. Aiguader, 88, 08003 Barcelona, Spain; 2 Universitat Pompeu Fabra (UPF), 08003 Barcelona, Spain

## Abstract

A novel model for the evolutionary origin of peroxisomes and related organelles from within the endoplasmic reticulum is proposed.

Peroxisomes and related particles—such as glyoxysomes and glycosomes—are found in a broad range of eukaryotes, and despite being functionally diverse, they have been shown to share common traits that point to a single evolutionary origin [1]. In particular, all members of the peroxisome organelle family share topogenic signals in organelle-targeted proteins and a core set of peroxins involved in the uptake of peroxisomal matrix proteins. The distribution of all these features in organelles of all the major eukaryotic groups indicates that a peroxisome-like organelle possessing such import machinery originated in the last common ancestor of all known eukaryotes. However, the specific nature of this evolutionary origin remains unknown, and has been the focus of much scientific debate [1–5]. Based on the broad diversity of enzymes found in at least one member of the peroxisome family, it was initially suggested that a common peroxisomal ancestor—the protoperoxisome—originally possessed all these enzymes and that the different types of peroxisomes evolved from this ancestor by selective, independent loss of particular enzymes, rather than by differential acquisition in each of the lineages [2]. An endosymbiotic origin of such a metabolically complex proto-peroxisome was also postulated based on the observations of remarkable parallelisms with organelles of clear endosymbiotic origin such as plastids and mitochondria [2]. For instance, some peroxisomal enzymes had a clear bacterial ancestry, peroxisomal proteins are synthesized in the cytosol and transferred post-translationally into the organelles under the guidance of certain specific targeting sequences, and new peroxisomes were found to originate by growth and division of existing ones.

Despite years of intense research efforts, no compelling evidence in support of the above-mentioned ideas has been obtained. Peroxisomes show no definitive sign of an endosymbiotic origin, as exists for mitochondria and chloroplasts, and phylogenetic data suggest an extremely diverse origin for the peroxisomal enzymes [1, 6]. Such diverse phylogenetic origin of metabolic enzymes in the peroxisomal matrix, as well as the increasingly diverse repertoire of metabolic pathways found in the peroxisomes of any species, argue against the endosymbiotic hypothesis and in favor of a model based on serial acquisition of enzymes rather than on differential loss. In addition, similarities with genuine bacterial-derived organelles were weakened after the discovery that peroxisomes lack a genome, and that peroxisomal proteins are imported by a system radically different from that found in plastids and mitochondria [7]. Finally, recent findings have shown that peroxisomes can be formed de novo with the involvement of the endoplasmic reticulum (ER) [8]. Based on these findings, the idea that peroxisomes have their evolutionary origin within the endomembrane system has been entertained (see [1] and references therein). However, such a hypothesis has never been formally articulated in the context of a plausible evolutionary scenario describing the possible selective forces involved. Here, I propose a new hypothetical model for the origin of peroxisomes in which they are considered evolutionary off-shoots of the endomembranous system, rather than a result of bacterial endosymbiosis. This scenario fits within the growing body of current data and has fundamental implications for our understanding of the origin and evolution of metabolic compartmentalization in eukaryotes.

At the start, I propose an initial phase of compartmentalization within a primitive endoplasmic reticulum in which some enzymes would be confined to specific areas of the organelle. Intracellular organization is a key factor in cellular metabolism [9, 10], and I envision that a plausible selective pressure for an increasing level of compartmentalization within the ER could have emerged from the advantage of ensuring physical proximity of enzymes involved in the same metabolic process. This is a common situation in modern ER, which consists of continuous but distinct subdomains devoted to specific functions such as secretion of proteins or lipid synthesis [11]. Numerous examples exist in which proteins are targeted to specific subdomains in modern ER. For instance, to minimize the mixing of prolamines and gluten, their mRNAs (and thus the synthesis of the proteins) are targeted to different ER subdomains [12]. Yet, another mechanism for segregating certain enzymes might be the existence of domains with specific lipid composition within the ER membrane [13]. Similar mechanisms of sub-compartmentalization within the ER may have been in place in the ancestral eukaryote where the peroxisome originated.

I further propose that certain reactions of the fatty acid metabolism were at the core of the evolutionary forces driving the separation of peroxisomes from the ER. This is based on the facts that (i) fatty acid metabolism is the most widespread and thus presumably the most ancient trait in peroxisomes and related organelles [1, 6]; and (ii) some of the oxidative enzymes that can be traced back to the ancestral peroxisomes [6] are involved in pathways, such as the synthesis of poly-unsaturated fatty acids that in some species require both ER and peroxisomal steps [14]. Furthermore, some of these enzymes are known to produce highly reactive species that are toxic to proteins, modifying them and causing inactivation. For instance, reaction of such compounds with the free thiols of cysteines leads to increasingly oxidized forms, from disulphide bonds to sulphinic, sulphenic, and sulphonic. The effects of highly reactive oxygen species are likely to be especially dangerous in the ER, where controlled oxidative folding of proteins occurs [15], thereby providing a strong selective force favoring any step towards the separation of these reactions from the ER.

An ancestral enzyme that presents all of these particularities is the H_2_O_2_-producing, peroxisomal acyl-CoA oxidase (Pox1p in yeast). Pox1p and its homologues participate in the early stages of beta-oxidation of long-chain fatty acids, and in some desaturation steps in the synthesis of polyunsaturated fatty acids [14]. This enzyme is widespread in eukaryotes, where it has a peroxisomal localization. Interestingly, the gene coding for this enzyme has been lost secondarily in organisms that are devoid of peroxisomes or related organelles such as apicomplexans and microsporidia [1], suggesting a strong evolutionary association between this enzyme and the organelle.

Hence, the complete separation of peroxisomes from the ER would have definitely precluded toxic interactions between lipid metabolism and other processes in the ER, leading to the formation of independent vesicles, the earliest peroxisomes or proto-peroxisomes. The acquisition by this ancestral membranous vesicle of a post-translational mechanism of protein import, and of at least one protein containing a targeting sequence recognized by this machinery, would have provided the proto-peroxisomes with a mechanism to gradually separate its biogenesis from the ER. This mechanism may have initially ensured that some toxic compounds were only produced when peroxisomes were detached from the ER. Interestingly, some recent studies point to the existence of mechanisms to ensure that the import machinery is only active when peroxisomes are fully separated from the ER [16]. In addition, the ability to incorporate freshly made proteins once the organelle was separated from the ER may have enabled mature peroxisomes to incorporate and maintain necessary quantities of enzymes according to the physiological needs. Earlier analyses have established an evolutionary link between the modern peroxisomal targeting system and the endoplasmic reticulum associated decay (ERAD) system [17, 18]. This similarity extends beyond sequence homology into how the two complexes function mechanistically [19]. Accordingly, an origin of the import machinery can be postulated that involved a duplication of the whole ERAD pathway and subsequent specialization to recognize and ubiquitinate Pex5p, the protein used to shuttle proteins into the organelle (Fig. 1).

**Fig. 1:**
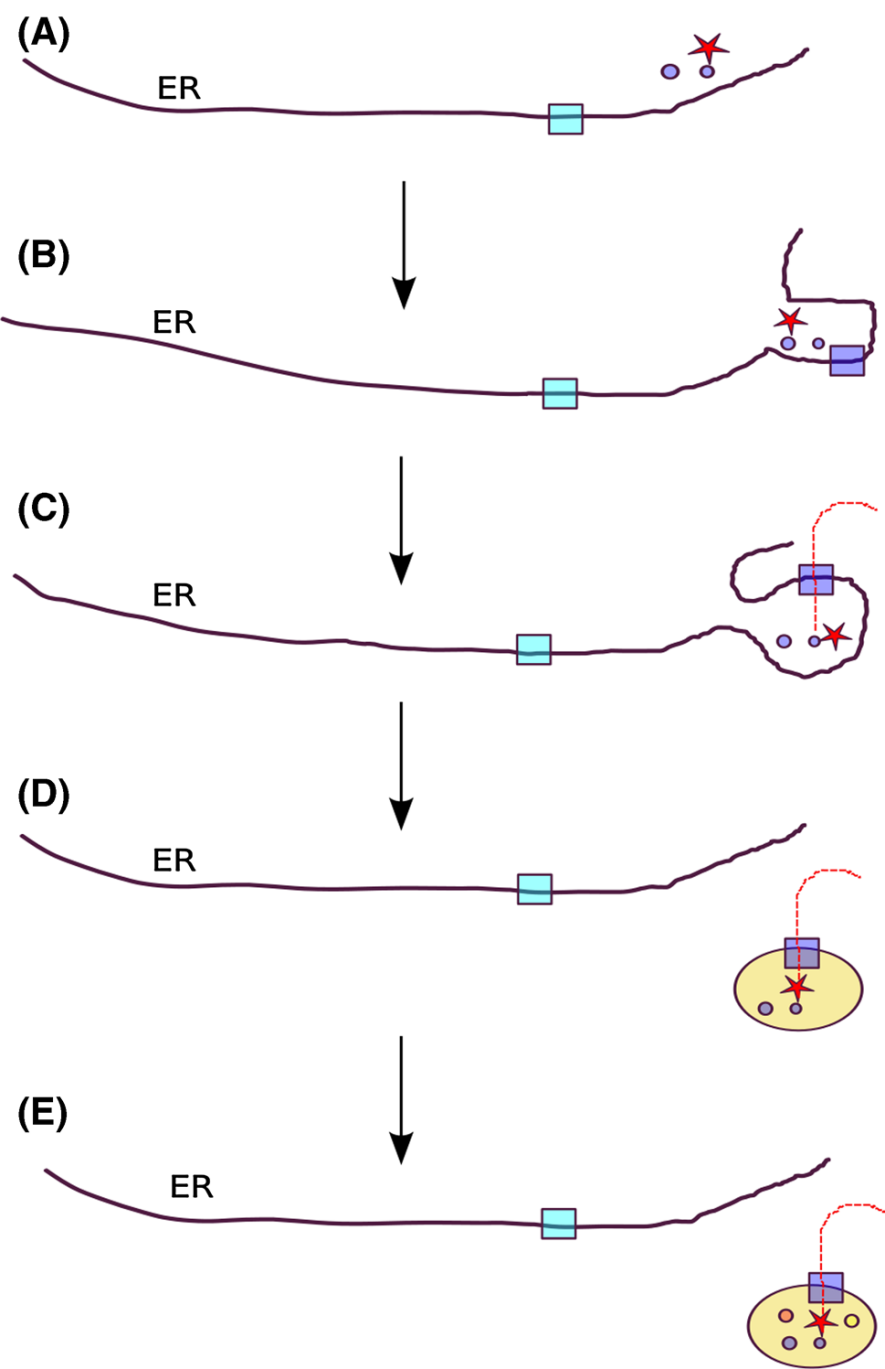
Schematic representation of a plausible scenario for the origin of peroxisomes from within the endoplasmic reticulum: **a** Some ER enzymes involved in lipid metabolism (*circles*) of which some produce toxic compounds (*red star*) are preferably localized in a specific region of the ER to minimize toxic effects on other ER functions. The ERAD system (*light blue square*) is in place and works by removing unfolded proteins from the ER. **b** The physical separation of the specific protoperoxisomal compartment increases, a duplication product of the ERAD system (*dark blue square*) is specifically localized to this sub-compartment. **c** Direct transport through an ERAD-derived translocation system enables safe import of the toxic-producing enzyme(s) to the proto-peroxisomal sub-compartment. **d** This compartment can eventually be detached from the ER and the matrix enzymes can be imported directly into the new organelle. **e** Acquisition of peroxisomal targeting signals by other genes enables the incorporation of novel enzymes of phylogenetically diverse origins leading to the diverse composition in the different lineages. Peroxisomes can be formed by division of pre-existing ones, since most of its components are translocated through the specific import machinery

Once the targeting and post-translational import machinery was in place, the interplay of genomic re-arrangements and other gene reshufflings in the nucleus may cause any number of new proteins to acquire the appropriate targeting sequence, just as it occurs with proteins targeted to mitochondria and plastids [20, 21]. The action of natural selection would have favored the retention of whatever such acquisitions that may have been advantageous under the surrounding conditions [22]. Such a mechanism could conceivably generate metabolically distinct peroxisomal lineages sharing only a common protein import machinery, depending on selective environmental conditions. Note that this mechanism also opens the door for varying the enzymatic content of already-formed peroxisomes within a single organism, according to the physiological needs. Just as it has been the case in the evolution of mitochondrial proteome [23], the origin of enzymes targeted to the peroxisomes in each lineage would have been diverse and based on various selective forces. An extreme case of adaptation, for instance, is that of the acquisition of glycolytic enzymes into the peroxisomes of kinetoplastids, some of these enzymes were probably acquired by means of horizontal gene transfer from bacteria [24]. According to reconstructions of the ancestral peroxisome present in the last common eukaryotic ancestor [6], catalase and some steps from the beta oxidation of fatty acids would have been among the first activities to be targeted to proto-peroxisomes. Interestingly, the origin one of these enzymes (Pox2p in yeast) can be traced back to the alpha-proteobacterial ancestor of mitochondria, suggesting the re-targeting of the beta oxidation of long-chain fatty acids to peroxisomes. In contrast, and in agreement with an origin within the ER, Pox1p has few homologues among alpha-proteobacteria and cannot be traced back to the ancestor of mitochondria [6]. Hence, in contrast to earlier suggestions [25], this model does not necessarily imply that mitochondrial endosymbiosis pre-dated the origin of peroxisomes. Note that coupling the re-targeting of mitochondrial enzymes to the formation of peroxisomes implies that these enzymes were targeted first to the ER, from where they would have promoted the formation of peroxisomes. The proposed model is thus simpler, and uncouples the origin of the two organelles, yet it does not provide information on which of the two appeared earlier.

Altogether, I argue in favor of a model in which peroxisomes are evolutionary off-shots of the endoplasmic reticulum. In face of existing data, I have proposed a plausible mechanistic scenario involving the avoidance of toxic by-products of the lipid metabolism as an initial driving force for the separation of the organelles, and the development of a targeting machinery in part derived from the duplication of the ERAD system. Once peroxisomes were autonomous organelles, the interplay between protein retargeting and selection shaped the existing metabolic diversity of peroxisomes in the different lineages. A testable prediction from this model is that Pox1p should remain among the most ancestral components of peroxisomes being lost only secondarily in some particular eukaryotic lineages. In addition, if a eukaryotic lineage is found that diverged prior to the origin of peroxisomes, the finding of a ER location of Pox1p homologues in such pre-peroxisomal eukaryotes will reinforce this model.
